# Anomalous right pulmonary artery from aorta, surgical approach case report and literature review

**DOI:** 10.1111/jocs.15618

**Published:** 2021-05-28

**Authors:** Khaled Alhawri, Ali Alakhfash, Abdullah Alqwaee, Mohammed HassabElnabi, Fazel Ahmed, Mohammed Alhawri, Bana Nasser, Marwan Alhoobani, Giusseppe Mazzesi, Abdulraoof Alsaeedi, Abdulrahman Almesned

**Affiliations:** ^1^ Prince Sultan Cardiac Center–Qassim Buraydah Saudi Arabia; ^2^ Department of General and Specialized Surgery ‘Paride Stefanini’ Dottorato di Ricerca La Sapienza, University of Rome Rome Italy; ^3^ International University of Malaya Wales Kuala Lumpur Malaysia; ^4^ King Fahad Armed Medical hospital Jeddah Makkah Saudi Arabia

**Keywords:** anomalous pulmonary artery from aorta

## Abstract

**Background:** Anomalous origin of one pulmonary artery from the aorta is a rare congenital anomaly affecting the right pulmonary artery more than the left. These patients are at risk for the early development of significant pulmonary hypertension. Early surgical treatment has been proven safe with excellent results. The surgical approach and technique is challenging and should be decided ahead before the patient to surgery. Different techniques were described including direct reimplantation, conduit interposition, aortic ring flap. **Aim:** We present a neonate with anomalous origin of the right pulmonary artery from the aorta and discuss the surgical technique and complications in the literature.

## 1 INTRODUCTION

Anomalous origin of one pulmonary artery from aorta is rare and was first described by Fraentzel in 1868. Direct aortic origin of the right pulmonary artery is much more common than the left one.^1^ This anomaly results in a large left‐to‐right shunt with the entire cardiac output from the right ventricle going to one lung while the other lung receives blood at systemic pressure from the aorta. Often other additional intracardiac shunting lesions are exacerbating the total hemodynamic burden. Thus the pulmonary vascular bed of both lungs may be vulnerable to the development of obstructive vascular disease.^2^

The term Hemitruncus is considered inadequate as per professor Anderson et al.^3^ because unlike—truncus arteriosus—two arterial valves aortic and pulmonary are present. About 300 cases have been reported in the literature, most of them in relation to surgical correction.^4^ Choosing the appropriate technique for repair depends on the site of origin of the anomalous pulmonary artery from aorta.

We describe a case of a neonate with this anomaly and discuss the possible surgical techniques and complications.

### 1.1 Ethics Statement

Approval from the institutional board review is available. In addition, an informed consent has been taken from the parents.

## 2 CASE PRESENTATION

**Figure 1:**
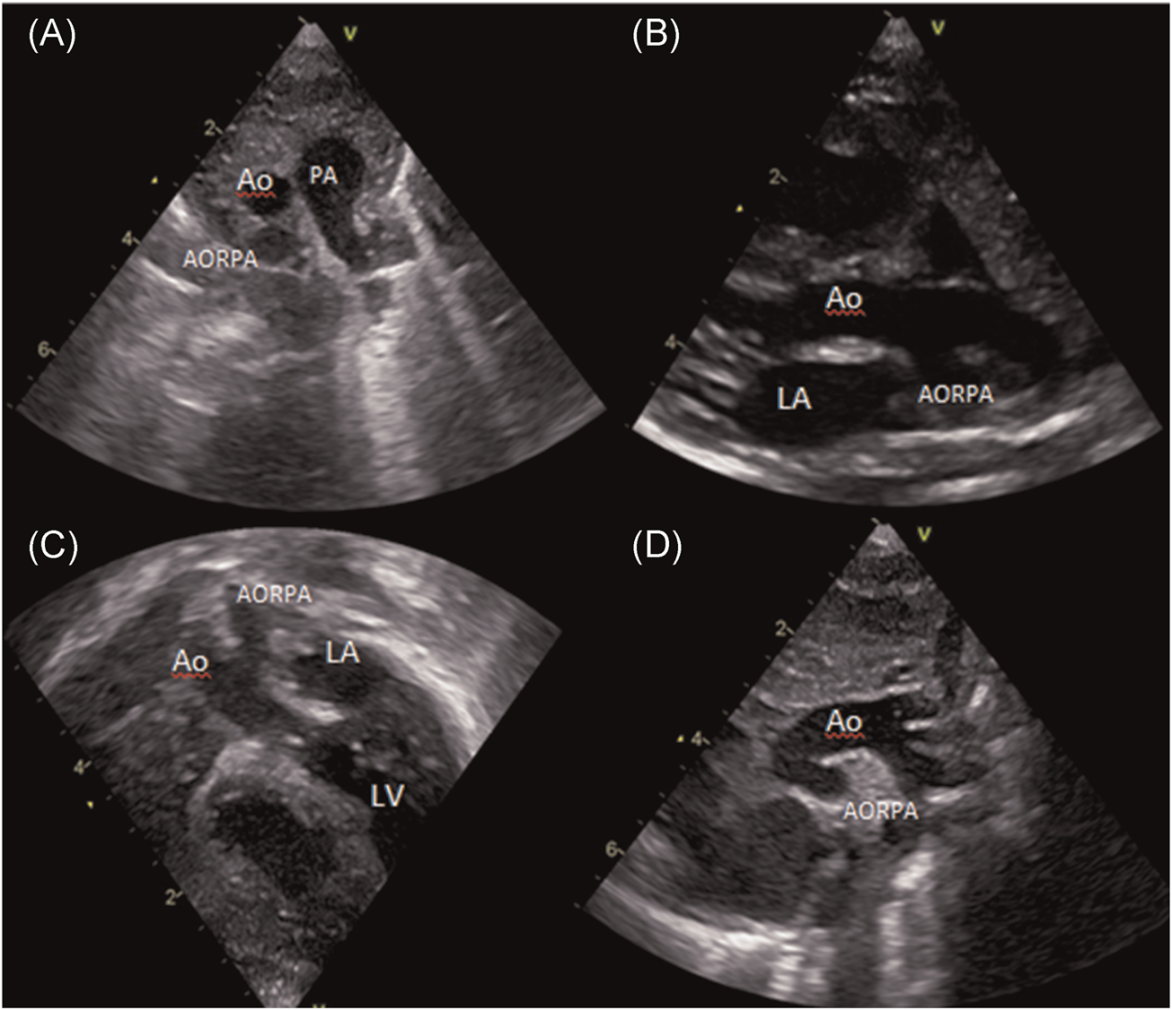
The preoperative echocardiogram. (A) Short‐axis parasternal view showing no continuity between the main pulmonary artery and the right pulmonary artery, (B) long‐axis parasternal view, (C) 5 chamber view, (D) suprasternal view all showing abnormal vessel coming out of the ascending aorta. Ao, aorta; AORPA, anomalous origin of right pulmonary artery from aorta; LA, left atrium; LV, left ventricle; PA, pulmonary artery

**Figure 2:**
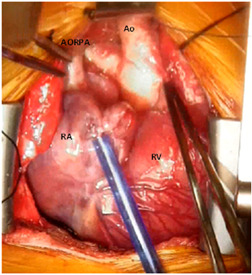
Operative view showing the anomalous origin of the right pulmonary artery from aorta. Ao, aorta; AORPA, anomalous origin of the right pulmonary artery from aorta; RA, right atrium; RV, right ventricle

**Figure 3:**
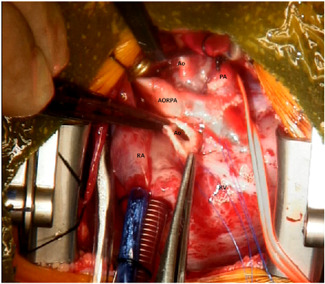
Operative view showing the reconstucted anomalous right pulmonary artery connected to the main pulmonary artery. Ao, aorta; AORPA, anomalous origin of the right pulmonary artery from aorta; PA, pulmonary artery; RA, right atrium; RV, right ventricle

**Figure 4:**
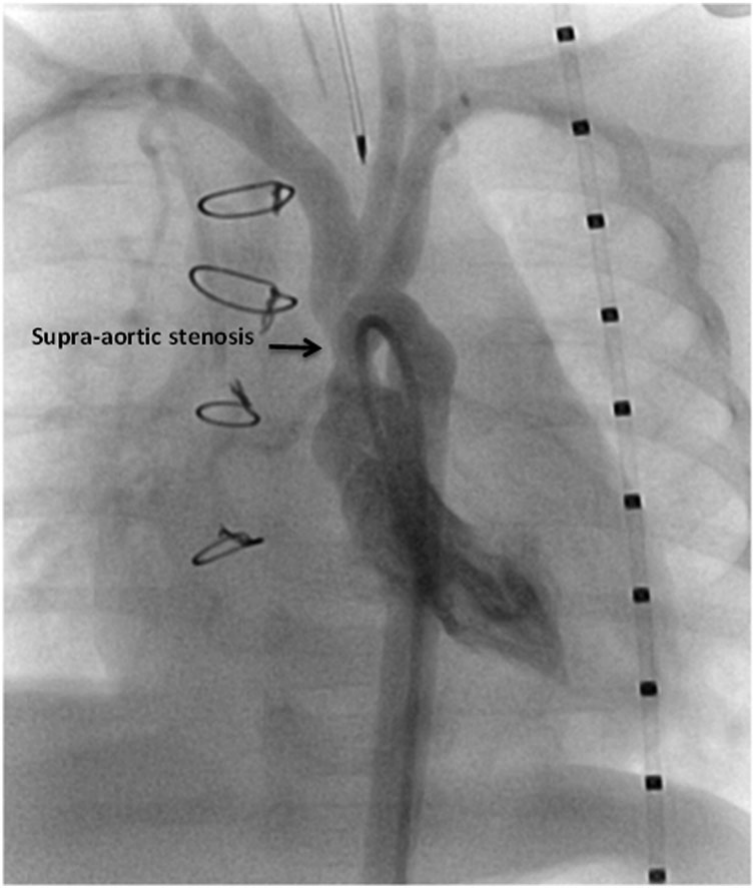
Anteroposterior cath view showing supra‐aortic stenosis at the site of anastomosis post repair

Our patient is a newborn female baby, weighing 3.3 kg, who was admitted to our neonatal intensive care unit as a case of anomalous origin of the right pulmonary artery from aorta (AORPA). She is a product of nonconsanguineous marriage and there is no history of congenital heart disease in the family. She was alert, well‐perfused but tachypnoeic. Her oxygen saturation was 94%, her chest X‐ray showed bilateral congested lungs. The echocardiogram (Figure 1) showed anomalous origin of the right pulmonary artery, left pulmonary artery from the main pulmonary artery with moderate size patent ductus arteriosus. No interventricular septal defect with small patent foramen ovale. Surgery was planned at Day 7 of life. However, the patient developed fever and leukocytosis (WBC: 33000, Platelet: 190000), so a septic workup was done and antibiotics were started and surgery was canceled to rule out sepsis. The patient needed elective intubation due to worsening tachypnea and desaturation to 85%. All septic screening was negative and the leukocytosis had improved. She was taken to the theater on Day 18 of life. The surgery was done under total cardiopulmonary bypass, aortic and single right atrial cannulation. Dissection around the ascending aorta till the neck branches, right pulmonary artery to the second branching (Figure 2), patent ductus arteriosus and left pulmonary artery to the second branching was completed. Ligation and division of the patent ductus arteriosus (PDA) were done to ensure good mobilization. The right pulmonary artery (RPA) was divided just above its entrance to the ascending aorta and a circular cuff (ring) was taken from the aorta after visualization of the coronary arteries origin and aortic valve leaflets, then the cuff was cut posteriorly to open the ring. An anterior flap was taken from the anterior main pulmonary artery and was used as a posterior wall for the RPA, and the aortic cuff was used as an anterior wall of the RPA. No patch was used to reconnect the right pulmonary artery to the main pulmonary artery (Figure 3). Then the aorta was reanastomosed anterior to the RPA. When we tried to wean the patient from bypass he developed desaturation and the right ventricle was distending, the pulmonary artery pressure was almost systemic. The echo showed significant obstruction to the flow across the main pulmonary artery. The pulmonary artery confluence was compressed by the ascending aorta. We went back on bypass and the right pulmonary artery was disconnected from the main pulmonary artery and brought anterior to the aorta (LeCompte maneuver) to avoid repeated ischemic aortic cross‐clamp. An autologous pericardial patch was used anteriorly to reconstruct RPA anastomotic site. This time the weaning off bypass was smooth with oxygen saturation of 100%, and pulmonary pressure of one‐third systemic. We kept the chest open prophylactically due to long bypass. In the intensive care unit, the patient had a smooth postoperative course. The chest was closed the next day. The patient was extubated on Day 3 postoperatively and discharged on the 8th postoperative day. One month later the patient presented with tachypnea. Echocardiography showed RPA significant osteal stenosis and supra‐aortic stenosis. RPA balloon dilatation was successful to reduce the pressure gradient. Aortic balloon dilatation was able to reduce the aortic mean pressure gradient from 90 to 40 mmHg (Figure 4). The case was discussed and agreed to go for surgical repair of the supra‐aortic and right pulmonary artery. During surgery, we found a significant intimal thickening in the supra‐aortic area at the anastomotic site. A longitudinal incision was made laterally on the ascending aorta extending from the noncoronary sinus until 1.5 cm above the stenotic area. An equine patch (The Matrix Patch™ Germany an equine pericardial patch) was used to repair the supra‐aortic stenosis. Another pericardial patch (The Matrix Patch™ Germany an equine pericardial patch) was used to repair the right pulmonary artery origin stenosis. The postoperative echocardiography showed minimal obstruction across the ascending aorta and relieved RPA stenosis. The patient did well after the surgery and was discharged on the 6th postoperative day.

## 3 DISCUSSION

Anomalous origin of one pulmonary artery from aorta is an unusual cardiac structural abnormality. Several theories have been offered in terms of morphogenesis. One suggesting an error in the development of cells migrating from the neural crest to the fifth or sixth aortic arches can be discounted simply because there is no fifth pharyngeal arch seen during normal development. Unequal partitioning of the “conotruncus” by eccentric coalescence of the right and left outflow tract ridges can similarly be discounted since the outflow cushions separate the intermediate and proximal parts of the developing outflow tract.^4^

Lesions such as aortopulmonary window, or an anomalous origin of the right pulmonary artery from the aorta, are intrapericardial. They are best explained on the basis of failure to close the embryonic aortopulmonary foramen, this in turn reflecting inadequate growth of the aortopulmonary septum from the dorsal wall of the aortic sac. Failure of the latter process explains well why patients with an aortopulmonary window are frequently found with the right pulmonary artery arising from the aortic side of the window. Eccentric growth, but subsequent fusion, of the aortopulmonary septum with the distal ends of the outflow cushions, provides a plausible explanation for the origin of the right pulmonary artery from the ascending aorta. The separate origins of the aortic and pulmonary roots in this lesion point to the inadequacy of using the term ‘‘hemitruncus’' for its description.^3^

Anomalous right pulmonary artery from the ascending arota is four to eight times more common than anomalous origin of LPA from aorta.^5^

AORPA is classified into two subgroups according to the morphological features: proximal and distal forms. In the proximal form, the anomalous pulmonary artery (PA) arises proximally from the posterior or left posterior aspect of the ascending aorta close to the aortic valve. About 85% AORPA is regarded as the proximal form in the report of Kutsche and Van Mierop. In the distal form, the anomalous PA originates from the ascending aorta just proximal to the innominate artery or from the base of the artery itself.^6^ Furthermore, the site of origin of the pulmonary artery can be frequently, ipsilateral (right or left lateral) or less commonly posterior.^7^

To diagnosis this anomaly, electrocardiography and chest X‐rays are important, but echocardiography is essential because the abnormal vessel pattern can be seen on two‐dimensional (2D) and Doppler examination. It is also important to distinguish this anomaly from discontinuous pulmonary arteries in which the blood supply to the left pulmonary artery originates from the major aortopulmonary collaterals or the ductus arteriosus.^8^

Computed tomography scan or magnetic resonance imaging can confirm the diagnosis. Many centers have also used catheterization whether to measure the PA pressure or for anatomical delineation (see the table). In our case, the patient was diagnosed by echo. Table 1 shows the diagnostic tools used in different centers across the world. Catheterization was used mainly to measure pulmonary artery pressure and pulmonary vascular resistance.

**Table 1:** Showing different centers experience with the management of anomalous origin of one pulmonary artery from aorta

Author	No. of cases	Ano. RPA	Ano. LPA	Method of diagnosis	Age at intervention (range)	Type of surgery	Morbidity	Associated defects	Mortality	Cause of death	Follow ‐up time	Reintervention	Comment
Abu‐suliman et al. (Canada)^9^	16	12	4	6 (echo) 10(cath)	30 d (1–3.3 y)	8 Reimp 5 Patch		6 PDA, 3ASD, 3VSD, 3 TOF, 2 COA, 1 ARSCA	3	3 intra‐operative (1 intra op before sx) 2 (with associated lesions ASD and VSD early in series)	16m–16y	1 (surgery) RPA augmentation 2 (cath RPA stent)	2 died before surgery (1 complex anatomy and 1 died before repair)
Peng et al. (UK)^10^	9	9	0	7 (echo) 2 (cath)	5 (under 30 d) 3(under 6 m) 1(1y)	8 Reimp 1 conduit(RV to RPA)		6 PDA, 3 PFO, 3 TOF, 2 ALSCA, 1 LSVC	0		7m–17y	2 (surgery) a. conduit replacement at 9 y b. RPA patch augmentation 1 ((cath RPA stent at 1 y after sx)	
Nathan et al.^2^ (USA)	16	14	2	12 (echo) 3(routine cath early in series) 1 (intra‐op LPA)	84 10 (under 30d) 4 (under 6 m) 1 (8 m) 1 (2 y)	11 Reimp 5Patch		13 PDA	1	5d old arch repair, vsd closure and TEF, died of sepsis	1–17 y	1 (surgery) supra AS and RPA stenosis surgical repair) 4 (cath) RPA balloon and stent)	
Kajihara et al.^6^ (Japan)	8	8	0	8 (echo) 6(cath for PAP)	35 mean (7–180d)	7 Reimp 1 PTFE	1 (ARF pt was sick pre op and sx done without bypass using PTFE 4) 1(pul. Hypertension crisis and CPR open cardiac massage then improved and discharged)	7 PDA, 1 ARSCA,1 COA,1 ASD	0		2m–13y	2 (surgery) a. supra AS surgicalrepair 3 y after repair b. RPA surgical patch augmentation 3 (Cath) RPA balloon)	
Erdem et al. (Turkey) ^11^	7	6	1	7 (echo) 2 (cath for associated defects e.g arch)	4–84	7 Rimp	2 (pulmonary hypertension needed prolonged intubation)	3 PDA, 1 IAA, 1 APW,	0		2m–6y	0 (only one pt had mild RPA stenosis for follow up)	
Amir et al.^12^ (Israel)	12	10	2	6 (echo) 6 (cath to confirm Dx)	49 (5–180)	8 Reimp 3 Patch 1 BT		12 PDA, 1 VSD, 2 ASD, 1 COA	1	5 m had severe hypoplastic RPA, BT shunt done and never done RPA repair due to PHT	1m–7y	3(cath RPA balloon)	
Talwar​ et al. (India)^13^	11	5	6	11 (cath) 4(CT)	73 m(7m–18y)	7 Reimp 1 Patch 1 conduit (saphenous)		9 TOF	2	1 post shunt with persistent hypoxia 1 low cardiac output	3–73 m	Non Pt post band awaiting total correction	All with TOF. All had total repair except 2 pt a. 1 RPA band with innominate LPA shunt b. 1 innominate to RPA shunt no bypass
Vasquez et al. (Mexico)^14^	5	3	2	4 (echo) 3 (CT) 2 (cath for PA Pressure)	(4m–10y)	5 Reimp		2 PDA, 1 ASD, 1 SubAS, 1 LPA stenosis	1	4 m taken as emergency due to desaturation, post operative had low cardiac output and died	2–6 m	Non	
Liu et al.^15^ (China)	11	7	4	Echo, CT, and cath variably	12.7(2m–3y)	6 Reimp 3 aortic ring 2 aortic flap		12 PDA, 1TOF, 11 ASD, 1ARSCA, 2 VSD, 1COA, 2 APW,	1	11 m with TOF due to low cardiac output	6–60 m	Non	No lecmopt used 2 pt had APW the window kept and used as falp fill space between RPA and MPA
Liu et al.^7^ (China)	19	17	2	19 (echo)19(CT)	3 m (4d–21y)	11 Reimp 1 Patch 2 aortic falp		4 PDA, 3 TOF, 4 VSD, 5 ASD, 2 APW, 1IAA			1–84 m	Non	5 pt non operated probably due to high operative risk due to PHT
Cho et al. (S. Korea)^16^	12	8	4	12 (echo) 6 (cath)5(CT)	(3–734d) 5 neonate	7 reimp 5Patch		9 PDA, 2 TOF, 2 COA, 6 ASD, 1 MAPCAS	1	Pt with multiple VSD and complete heart block post and ECMO and PHT crisis	1–12 y	1 (surgery) 14 y post op LPA augmentation 2 (cath) RPA balloon	

Abbreviations: Ano. RPA, anomalous origin of the right pulmonary artery from aorta; Ano. LPA, anomalous origin of the left pulmonary artery from aorta; APW, aortopulmonary window; ALSCA, aberrant left subclavian artery; ARF, acute renal failure; ARSCA, aberrant subclavian artery; ASD, atrial septal defect; BT, Blalock‐Taussig; CT, computed tomography; COA, coarctation; CPR, cardiopulmonary resuscitation; d, days; IAA, interrupted aortic arch; LSVC, left superior vena cava; m, month; MAPCA, major aortopulmonary collaterals; Patch, patch augmentation; MPA, main pulmonary artery; PDA, patent ductus arteriosus; PHT, pulmonary hypertension; PTFE, polytetrafluoroethylene; Reimp, direct reimplantation; RPA, right pulmonary artery; RV, right ventricle; SubAS, sub aortic stenosis; Sx, surgery; TEF, tracheosephageal fistule; TOF, tetralogy of fallot; VSD, ventricular septal defect; y, years.

Associated anomalies include PDA, ventricular septal defect (VSD), tetralogy of Fallot, interrupted aortic arch, aortopulmonary window, isthmic hypoplasia, and aberrant right subclavian artery. DiGeorge syndrome is less common with hemitruncus anomalies as compared with other conotruncal malformations such as truncus arteriosus or interrupted aortic arch.^5^

The most commonly associated anomaly is PDA.^6^ This is evident in Table 1.

The natural history of this condition is characterized by the onset of severe pulmonary vascular obstructive disease (PVOD) if not corrected early. This anomaly results in a large left‐to‐right shunt with the entire cardiac output from the right ventricle going to one lung while the other lung receives blood at systemic pressure from the aorta. Often other additional intracardiac shunting lesions are exacerbating the total hemodynamic burden. Thus the pulmonary vascular bed of both lungs may be vulnerable to the development of vascular disease. If left untreated, 1‐year survival has been reported to be as low as 30%.^2^ Moreover, coronary ischemia, (steal syndrome), due to systemic diastolic blood flow to the anomalous pulmonary artery and PDA, has been reported.^10^

Amir et al. discussed their experience with this anomaly and compared the pre and postoperative right ventricular pressure in these 12 patients. All but one patient had normal right ventricular pressure postoperatively. That patient had hypoplastic RPA supplied by closed PDA. Blalock‐Taussig shunt was done to promote the RPA growth but the patient had a recurrent chest infection and high pulmonary pressure in the LPA and eventually died at 3 months after surgery when he was 5 months old.^12^

Many centers have described their experience with this anomaly as case reports or case series. Table 1 summarize some of the published series with a special note on the number of cases, which side of pulmonary artery abnormal branching, the associated defects, the technique of repair, mortality and morbidity, and reintervention.

### 3.1 Timing of surgery

Surgery should be offered as soon as the diagnosis is established. Congestive heart failure, the development of progressive pulmonary vascular disease and early death can start as early as 3 months of age. Gupta et al.^17^ reported a successful attempt to restore fetal circulation by the use of prostaglandin (PgE1), aiming to open the ductus and decompress the right ventricle as a temporary bridge to preparation for corrective surgery. Surgical correction in the neonatal period provides excellent short‐ and long‐term outcomes. The neonatal correction has increased dramatically over the past two decades.^4^, ^9^ Moreover, repair in a preterm 1.6 kg, 34‐week‐gestation baby has been reported.^18^ Adults presentation has been reported and surgery was abandoned due to the high risk related to the severe pulmonary hypertension. Yet, successful repair has been reported in adults.^19^, ^20^, ^21^ The presence of associated complex cardiac defect increases the risk of surgery and should be assessed individually.

### 3.2 Surgical technique

Historically PA banding, PDA ligation alone, or PDA ligation with ligation of the RPA were described and were unsuccessful.^22^ Penkoske and associates reported that the mortality of this malformation with palliative operation including ligation of the anomalous RPA, ligation of the patent ductus arteriosus, or PA banding reached 82%.^23^

### 3.3 Cardiopulmonary bypass conduct

Aortic cannulation should be done as distal as possible, some surgeons advise doing it in the aortic arch to give enough length in the ascending aorta for cross‐clamp and aortic reconstruction. Although there is no technical reason to use deep hypothermia with either low flow cardiopulmonary bypass (CPB) or circulatory arrest, some surgeons prefer it. The branch pulmonary arteries are exposed and temporarily occluded with either nontraumatic microvascular clips or Silastic snares immediately after CPB is established to prevent flooding the lungs.^24^ If an aortic cross‐clamp is applied the anomalous pulmonary artery is kept occluded while giving the cardioplegic solution to arrest the heart.

Many methods have been described depending on the site of origin of the anomalous pulmonary artery.

**Figure 5:**
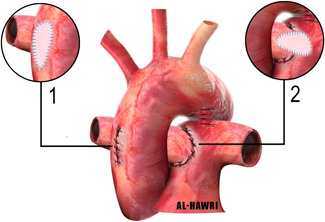
Direct implantation technique without and with patch augmentation of the aorta (inset 1), and patch augmentation of the right pulmonary artery (inset 2)

**Figure 6:**
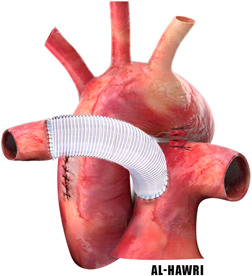
Conduit interposition of the anomalous right pulmonary artery using Dacron conduit graft

**Figure 7:**
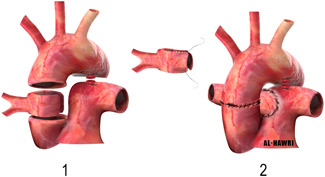
Use of aortic ring to elongate the RPA (single aortic flap technique)

**Figure 8:**
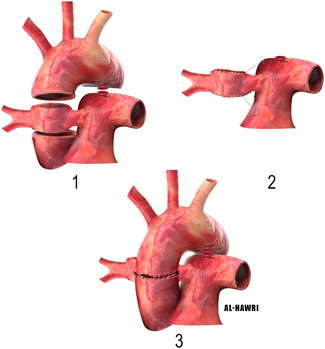
Use of aortic flap to simultaneously repairing AORPA and aortopulmonary window. AORPA, anomalous origin of the right pulmonary artery from aorta

**Figure 9:**
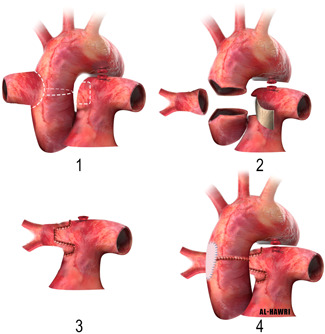
Double flap technique (ascending aortic and main pulmonary flaps), an anterior main pulmonary artery flap, and posterior aortic flap

**Figure 10:**
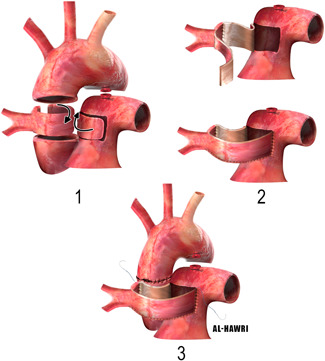
Double flap technique (ascending aortic and main pulmonary flaps), a posterior main pulmonary artery flap, and anterior aortic flap

1. Primary direct implantation with or without patch augmentation (Figure 5):
  This method was described first by Kirkpartick et al. in 1967. The RPA is disconnected and then direct implantation of the RPA to the side of the main pulmonary artery main pulmonary artery (MPA), with or without patch augmentation of the defect in the ascending aorta. Some patients were operated on without cardiopulmonary bypass. Some advocated using interrupted everting sutures anteriorly.^25^, ^26^
  Table 1 shows that this was the most common method used in different centers.
  Nathan et al. described successful repair in 13 out of 16 patients using direct implantation. They reported that the origin of RPA in these 13 patients was posterior while the other 3 patients had their RPA originated from the anterolateral aspect of the ascending aorta, in which they needed to use an autologous pericardial patch to augment the pulmonary anastomosis site.^2^ Li Xie et al. reported good results of six patients using direct implantation technique with no further reintervention needed postoperatively. Considering the growth potential of the pulmonary artery and the prevention of anastomotic obstruction, the direct implantation‐direct anastomosis of the anomalous pulmonary artery to the pulmonary trunk—seems to be the preferred procedure if it can be achieved without tension.^27^
  Primary repair with direct implantation is generally reserved for those patients in whom the RPA originates from the posterior aspect of the aorta in close proximity to the MPA.^28^
2. Use of interposition graft conduit (Figure 6):
  It was first described by Armer et al. in 1961 (Fontana 14). The technique is to use a graft conduit (e.g., Dacron Woven conduit) to fill the space between the RPA and MPA.^22^
  The drawback is that the patient will outgrow the conduit and will need reintervention.
3. Use of aortic ring to elongate the RPA (single aortic flap technique) (Figure 7):
  The ascending aorta is transected just above and below the origin of the AORPA to form an aortic ring, which is used to elongate the anomalous RPA. The RPA, aorta, and branches should be adequately mobilized. The aorta is then reconstructed anterior to the RPA as an end‐to‐end anastomosis.^29^
  There is always a risk of compression of the aorta on the pulmonary artery especially if it originates laterally. In that case, LeCompte maneuver with generous dissection of the ascending aorta and its neck branches in addition to the branch pulmonary arteries bilaterally might be useful.
4. Use of aortic flap to simultaneously repairing AORPA and APW (Figure 8):
  If there is an associated Aortopulmonary window, the APW is kept in place, as it could serve as a communication between the MPA and aberrant RPA. The ascending aorta, proximal aortic vessels, MPA, and aberrant RPA are mobilized widely. The ascending aorta is transected around the origin of the aberrant RPA and APW with an aortic flap connected to the MAP and aberrant RPA. The aortic flap is used to reconstruct a conduit between the MAP and anomalous RPA. Then, an end‐to‐end anastomosis of the aorta is performed in front of the RPA. A patch can be used to augment the RPA anteriorly.^15^
5. Double flap technique (ascending aortic and main pulmonary flaps):
  a. An anterior MPA flap (Figure 9):
    Van Son and Hanley in 1996 described an anterior MPA flap anastomosis to a posterior aortic/branch pulmonary arterial flap. They transected the aorta transversely with a generous cuff around the RPA, then an anterior hinge trap door incision is made in the MPA, opposite the LPA, to make an anterior pulmonary flap to create a balance against the posterior aortic flap on the RPA. Finally, the aorta is anastomosed directly anterior to the RPA.^30^
  b. A posterior MPA flap (Figure 10):
    Another way to do the double flap technique is by taking an aortic ring cuff with a diameter 1.5 times more than the size of the RPA, then this cuff is cut so as to leave a small posterior aortic flap. MPA flap is used as a posterior flap. LeCompte maneuver is used to bring the RPA anterior to the aorta to avoid aortic compression.

Prifti et al., reported this method as a modification of Van Son et al. technique. They justified this by saying, an end‐to‐end anastomosis of the ascending aorta will retract it more posteriorly, which may induce the possibility of later obstruction of the newly created RPA placed posterior to the aorta due to aortic compression.^31^

We adopted the double flap technique in our case because the RPA originated from the right lateral of the aorta and not posterior, making the RPA away from the MPA. However, we did not use the LeCompte maneuver initially until we had to go back on bypass as we found the right ventricular pressure is almost systemic due to obstruction in the main pulmonary artery flow related to the ascending aortic compression. After the LeCompte maneuver, the right ventricular pressure went down dramatically after that with smooth weaning off bypass. This could be related to the amount of tissue resected from the ascending aorta which can shorten it, and make it easy to compress the underneath right pulmonary artery to pulmonary artery anastomosis.

Hospital mortality has been reported as anywhere from 0% to 21%, and the need for reintervention, 12.5% to 36%.^2^, ^10^, ^32^, ^33^

Table 1 shows the mortality in different centers experience with the cause of death detailed.

Echocardiography is useful in the postoperative follow‐up. Catheterization can be performed to further diagnose or treat any residual lesion when needed.

Our patient developed RPA osteal stenosis and needed balloon dilatation at 1 month of age. This could be related to the fact that the anastomosis was repeated to do the LeCompte maneuver. She also developed stenosis at the site of aortic anastomosis. The Balloon dilatation was not successful enough for that and the patient was sent for surgical repair when she was 5 months old.

Nathan et al. reported the complication of supra‐aortic stenosis postoperatively.^2^ Kajihara et al. recommended the augmentation of the aortic root defect with a patch whenever even slight supra‐aortic stenosis is suspected.^6^ In our patient, the ascending aorta was small and due to some tension, we have added extra stitches to control the bleeding. This could be the cause of supra‐aortic stenosis in our patient. During surgery, we found a significant intimal thickening from within although the anastomosis did not show any hourglass appearance from outside.

## 4 CONCLUSION

We conclude that the anomalous origin of one pulmonary artery is a dismal but surgically correctable disease. Careful diagnosis and timing of the operation is of utmost importance as pulmonary hypertension can start early in life. The surgical technique has to be planned preoperatively depending on the site of origin of the branch pulmonary artery from the aorta. Direct implantation is the commonest method used in the literature unless there is a lateral origin of the branch PA, which mandate different technique like patch augmentation or aortic ring. The use of an aortic ring with pulmonary artery anterior flap, flipping it as a posterior wall of the anastomosis, is a useful technique when the RPA originate from the right anterolateral aspect of the ascending aorta. LeCompte maneuver, with good branch pulmonary arteries peripheral dissection to the second bifurcation, is a useful technique to avoid direct compression of the ascending aorta on the anastomosis when using this technique.

## CONFLICT OF INTERESTS

The authors declare that there are no conflict of interests.

## AUTHOR CONTRIBUTIONS

**Khaled Alhawri**: Corresponding author, drafting and revision; **Ali Alakhfash**: Revision and editing; **Abdullah Alqwaee**: Revision of the echo and cath views and diagnosis; **Mohammed H. Elnabi**: Drafting article; **Fazel Ahmed**: Drafing article. **Mohammed Alhawri**: Drawing the figures; **Bana Nasser**: Collecting data and revision; **Marwan Alhoobani**: Collecting data and revision; **Giusseppe Mazzesi**: Critical revision; **Abdulraoof Alsaeedi**: Revision and concept; **Abdulrahman Almesned**: Concept of paper.
